# The Norwegian tenecteplase stroke trial (NOR-TEST): randomised controlled trial of tenecteplase vs. alteplase in acute ischaemic stroke

**DOI:** 10.1186/1471-2377-14-106

**Published:** 2014-05-15

**Authors:** Nicola Logallo, Christopher E Kvistad, Aliona Nacu, Halvor Naess, Ulrike Waje-Andreassen, Jörg Asmuss, Anne Hege Aamodt, Christian Lund, Martin W Kurz, Ole Morten Rønning, Rolf Salvesen, Titto T Idicula, Lars Thomassen

**Affiliations:** 1 Center for Neurovascular Diseases, Haukeland University Hospital, Bergen, Norway; 2 Department of Clinical Medicine, University of Bergen, Bergen, Norway; 3 Centre for Age-related Medicine, Stavanger University Hospital, Stavanger, Norway; 4 Centre for Clinical Research, Haukeland University Hospital, Bergen, Norway; 5 Department of Neurology, Oslo University Hospital, Rikshospitalet, Norway; 6 Department of Neurology, Stavanger University Hospital, Stavanger, Norway; 7 Medical Division, Department of Neurology, Akershus University Hospital, Lørenskog, Norway; 8 Department of Neurology, Nordland Hospital, Bodo, Norway; 9 Department of Clinical Medicine, University of Tromso, Norway; 10 Department of Neurology, St.Olav Hospital, Trondheim, Norway

**Keywords:** Acute ischaemic stroke, Alteplase, Intravenous thrombolysis, Tenecteplase

## Abstract

**Background:**

Alteplase is the only approved thrombolytic agent for acute ischaemic stroke. The overall benefit from alteplase is substantial, but some evidence indicates that alteplase also has negative effects on the ischaemic brain. Tenecteplase may be more effective and less harmfull than alteplase, but large randomised controlled phase 3 trials are lacking. The Norwegian Tenecteplase Stroke Trial (NOR-TEST) aims to compare efficacy and safety of tenecteplase vs. alteplase.

**Methods/Design:**

NOR-TEST is a multi-centre PROBE (prospective randomised, open-label, blinded endpoint) trial designed to establish superiority of tenecteplase 0.4 mg/kg (single bolus) as compared with alteplase 0.9 mg/kg (10% bolus + 90% infusion/60 minutes) for consecutively admitted patients with acute ischaemic stroke eligible for thrombolytic therapy, i.e. patients a) admitted <4½ hours after symptoms onset; b) admitted <4½ hours after awakening with stroke symptoms c) receiving bridging therapy before embolectomy.

Randomisation tenecteplase:alteplase is 1:1. The primary study endpoint is favourable functional outcome defined as modified Rankin Scale 0–1 at 90 days. Secondary study endpoints are: 1) haemorrhagic transformation (haemorrhagic infarct/haematoma); 2) symptomatic cerebral haemorrhage on CT 24–48 hours; 3) major neurological improvement at 24 hours; 4) recanalisation at 24–36 hours; 5) death.

**Discussion:**

NOR-TEST may establish a novel approach to acute ischaemic stroke treatment. A positive result will lead to a more effective, safer and easier treatment for all acute ischaemic stroke pasients.

NOR-TEST is reviewed and approved by the Regional Committee for Medical and Health Research Ethics (2011/2435), and The Norwegian Medicines Agency (12/01402). NOR-TEST is registered with EudraCT No 2011-005793-33 and in ClinicalTrials.gov (NCT01949948).

## Background

Alteplase is the only approved treatment for acute ischaemic stroke within 4½ hours after stroke onset. The overall benefit from alteplase is substantial, but up to 2/3 of patients with large artery occlusions may not achieve recanalisation and less than half of the patients treated, have complete reperfusion by 24 hours [1]. Up to 40% of thrombolysed stroke patients may therefore remain severely disabled or die [2], leaving substantial room for improvement. A growing body of evidence indicates that alteplase also has negative effects on the ischaemic brain, including cytotoxicity and increased permeability of the blood–brain–barrier facilitating cerebral edema [3-6]. An alternative thrombolytic therapy that might be easier and safer to administer could lead to wider acceptance and use of thrombolytic therapy [7]. Tenecteplase may be the drug of choice [8]. Tenecteplase is a modified tissue plasminogen activator that is more fibrin-specific, more resistant to plasminogen activator inhibitor (PAI), has a longer half-life than alteplase and can therefore be administered as an intravenous bolus [1,7,9].

In animal models of acute ischaemic stroke, tenecteplase results in more rapid and complete reperfusion, and less hemorrhagic conversion of experimental infarcts than equipotent doses of alteplase [10,11].

Several clinical trials of patients with myocardial infarction have shown that tenecteplase induces more rapid coronary reperfusion, with similar mortality rates, but significantly reduced rate of major bleeds as compared with alteplase [1,9,12].

In a dose-escalation safety study of tenecteplase, no symptomatic intracranial haemorrhages (SICH) were found among 88 ischaemic stroke patients treated with doses ranging from 0.1 mg/kg to 0.4 mg/kg [9].

A randomised study of tenecteplase (0.4 mg/kg) vs. standard alteplase in stroke patients (n = 122) with MCA occlusion and perfusion lesion ≥20% greater than the infarct core has shown that tenecteplase (0.4 mg/kg) leads to faster and more complete MCA 2 h-recanalization, better 24 h improvement, and better long-term outcome, without increased risk of cerebral haemorrhage [13].

A dose-ranging study of tenecteplase for patients with acute ischemic stroke, using standard clinical selection criteria, showed that a dose of 0.4 mg/kg was associated with excess SICH, whereas no difference was found between doses of 0.1 mg/kg and 0.25 mg/kg. Due to SICH and slow enrollment, the trial was stopped prematurely [7].

In a recently published randomised trial of tenecteplase 0.1 mg/kg (n = 25), tenecteplase 0.25 mg/kg (n = 25) or alteplase 0.9 mg/kg (n = 25) within a 6 hour time window and using CT-perfusion (mismatch) and CT-angiography (visible occlusion) as imaging entry criteria, tenecteplase was associated with better reperfusion, less SICH and better clinical outcome than alteplase [14].

Wake-up stroke (WUPS) patients are not eligible for thrombolytic treatment because the time of onset is not known. Patients with an acute ischaemic lesion detected with DW-MRI but not with FLAIR imaging are likely to be within a time window in which thrombolysis is safe and effective [15]. Intravenous thrombolysis with alteplase seems to be an independent determinant of a better outcome in WUPS and associated with a 5-fold odds of achieving an mRS 0 to 2 at 90 days without increasing the risk of haemorrhage [16]. The effect of tenecteplase in WUPS patients has not been studied.

Embolectomy recanalises large artery occlusions more effectively and rapidly than intravenous alteplase alone. Intravenous alteplase is routinely administered prior to endovascular treatment as a “bridging therapy”. There is no published evidence indicating an increased risk of combining tenecteplase with endovascular treatment.

### Aims

Aim of the Norwegian Tenecteplase Stroke Trial (NOR-TEST) is to compare efficacy and safety of tenecteplase 0.4 mg/kg (single bolus) vs. alteplase 0.9 mg/kg (10% bolus + 90% infusion/60 minutes) given a) <4½ hours after symptom onset, b) <4½ hours after awakening with stroke symptoms, and c) as bridging therapy before embolectomy <6 hours after symptom onset.

## Methods/Design

### Study design

NOR-TEST is a multi-centre PROBE (prospective randomised, open-label, blinded endpoint) trial, designed to establish superiority of tenecteplase as compared with alteplase for consecutively admitted patients with acute ischaemic stroke treated a) within 4½ hours after the onset of symptoms, b) within 4½ hours after awakening with symptoms, and c) within 6 hours after the onset of symptoms if eligible for embolectomy. Randomisation tenecteplase:alteplase is 1:1. The randomisation procedure uses envelopes in the thrombolysis-medication box to reduce time delay.

### Sample size

Power calculation was based on data from the following studies: pooled analysis of alteplase trials (n = 463) [17], SITS-MOST (n = 10.772) [18,19], the Bergen NORSTROKE 2007–2011 (n = 134) [i.e. alteplase in routine practice] and Parsons 2012 (n = 75) [i.e. tenecteplase RCT] [14].

NOR-TEST aims to detect a 9% higher percentage excellent outcome with tenecteplase vs. alteplase (r_1_ = 0.40; r_2_ = 0.49; OR 1.44; power 0.8), which requires inclusion of 954 patients (see Additional file 1). The study was initiated at Haukeland University Hospital, Bergen, in September 2012 and at the other centres during 2013 and January 2014. NOR-TEST is expected to be ended in autumn 2016.

### Patient recruitment

All patients found eligible for routine thrombolytic therapy are eligible for NOR-TEST. Inclusion criteria for thrombolytic therapy are defined as follows:

•Age 18 years or older.

•Ischaemic stroke with measurable deficit on National Institute of Health Stroke Scale (NIHSS).

•All ischaemic stroke sub-types and vascular distributions. A visible arterial occlusion is not required.

•Treatment within 4½ hours of stroke onset.

•Informed written consent signed by the patient, or verbal consent from the patient as witnessed by a non-participating health care person, or consent by the signature of the patient's family must be provided before treatment. Patients for whom no informed consent can be obtained will not be included in the study, but will be treated according to standard guidelines.

Specific sub-set inclusion criteria

•Wake-Up Stroke: Treatment within 4½ hours after awakening based on FLAIR-DWI mismatch on MRI [15].

•Embolectomy: Occlusion of cerebral intra-arterial vessel technically accessible for endovascular embolectomy <4½ hours after stroke onset or awakening; or <6 hours if based on penumbra assessment (as defined by local treatment protocols).

Exclusion criteria for thrombolytic therapy are defined as follows:

•Patients with premorbid modified Rankin Scale (mRS) score ≥3.

•Patients for whom a complete NIH Stroke Score cannot be obtained.

•Hemiplegic migraine with no arterial occlusion on baseline CT.

•Seizure at stroke onset and no visible occlusion on baseline CT.

•Intracranial haemorrhage on baseline CT.

•Clinical presentation suggesting subarachnoid haemorrhage even if baseline CT is normal.

•Large areas of hypodense ischaemic changes on baseline CT.

•Patients with primary endovascular treatment.

•Patients with systolic blood pressure >180 mm Hg or diastolic blood pressure >110 mm Hg despite of blood pressure lowering therapy.

•Pregnant or breast feeding.

•Known bleeding diathesis; use of oral anticoagulants and INR ≥1.4; heparin <48 hours and increased APTT; low molecular weight heparin(oid) <24 hours; new oral anticoagulants <12 hours; any other investigational drug <14 days.

•Sepsis, endocarditis.

•Patients with arterial puncture at a noncompressible site or lumbar puncture <7 days; major surgery or serious trauma <14 days; gastrointestinal or urinary tract hemorrhage <14 days; clinical stroke <2 months; history of intracranial haemorrhage; CNS neurosurgery <2 months; serious head trauma <2 months; pericarditis; any serious medical illness likely to interact with treatment; confounding pre-existent neurological or psychiatric disease; unlikely to complete follow-up.

### Per protocol exclusion after randomisation

The Norwegian Sonothrombolysis in Acute Stroke Study (NOR-SASS) [20] is a randomised study of Sonothrombolysis vs. sham Sonothrombolysis, superimposed on the NOR-TEST trial. Patients who receive NOR-TEST treatment (randomisation 1) and thereafter receive NOR-SASS sonothrombolysis (randomisation 2) are per protocol removed from NOR-TEST. Patients receiving sham sonothrombolysis are kept within NOR-TEST.

### Acute treatment and Stroke Unit care

Patients are treated according to the Departments’ Standard Operating Procedures (SOP) and will receive (state-of-the-art) multidisciplinary stroke unit care. Systolic blood pressure >180 mm Hg and/or diastolic blood pressure >110 mm Hg is treated with iv bolus of labetalol (Trandate®) 50 mg (10 ml) followed by infusion of labetalol as appropriate.

Thrombolytic treatment must start <4½ hours after symptom onset. Alteplase is given in a dose of 0.9 mg/kg (10% bolus + 90% infusion/1 hour), maximum dose 90 mg. Tenecteplase is given in a dose of 0.4 mg/kg as bolus, maximum dose 40 mg.

Endovascular treatment with mechanical embolectomy is initiated within 4½ hours after symptom onset if an accessible arterial occlusion is identified, or within 6 hours if based on penumbra assessment (as defined by local treatment protocols). The choice of embolectomy device is left to the experience and competence of the investigator. The use of conscious sedation or general anaesthesia during the endovascular procedure is registered.

If an anaphylactoid reaction occurs with alteplase or tenecteplase, treatment will immediately be stopped and appropriate anaphylactoid treatment given according to the Departments SOP. If an intracranial bleeding is clinically suspected, alteplase will immediately be stopped (tenecteplase is already given as a bolus) and an emergency CT will be performed. Bleedings will be treated according to SOP.

### Work-up

Neurological deficits are documented with National Institutes of Health Stroke Scale (NIHSS). Function is documented with mRS and Barthel Index (BI).

On admission, native CT with CT angiography (CTA) (or MRI/MRA) is performed. Baseline native CT is assessed using the Alberta Stroke Program Early CT score (ASPECTS) [21]. Intracranial MCA occlusions are trichotomized in none, distal (MCA_2_), or proximal (MCA_1_) occlusion. Other occlusions are defined as appropriate. MRI/MRA (or CT/CTA) is repeated after 24–36 hours to verify and assess infarction, assess intracranial recanalisation and to detect haemorrhagic transformations. Radiological assessment is performed by investigators blinded to type of thrombolysis. Long-term follow-up is performed as an Outpatient Clinic visit or by telephone interview at 3 months by a blinded and certified stroke nurse.

Patients excluded from NOR-TEST within the therapeutic time window of 4½ hours are also to be assessed clinically, and as far as possible also radiologically, in order to make an analysis of centre differences in inclusion/exclusion possible.

### Clinical outcome (neurology and function)

Early clinical outcome is defined by NIHSS score at 2 hours (NIHSS_2_) and 24–36 hours (NIHSS_24_) and assessed in an unblinded fashion by the investigator:

•Major Neurological Improvement: NIHSS = 0 or reduction of ≥4 NIHSS points compared with baseline (NIHSS_0_).

•Absolute reduction in NIHSS: NIHSS = ΔNIHSS_0_–NIHSS_2_ and ΔNIHSS = NIHSS_0_–NIHSS_24_.

Short term functional outcome (day 7, or earlier if earlier discharge) is assessed in a blinded fashion by a trained and certified stroke nurse or neurologist and defined by mRS score:

•Sliding dichotomy/responder analysis: Excellent outcome is defined as mRS 0 with baseline NIHSS ≤7, as mRS 0–1 with baseline NIHSS 8–14, as mRS 0–2 with baseline NIHSS ≥15.

Long term functional outcome (day 90) is defined at by mRS score:

•Fixed dichotomy: Excellent (mRS 0–1) vs. unfavourable outcome (mRS 2–6) (*primary study endpoint*).

•Fixed dichotomy: Good (mRS 0–2) outcome vs. bad outcome (mRS 3–6).

•Sliding dichotomy/responder analysis: Excellent outcome defined as mRS 0 with baseline NIHSS ≤7, as mRS 0–1 with baseline NIHSS 8–14, as mRS 0–2 with baseline NIHSS ≥15.

•Single mRS group comparison.

Final follow-up at 90 days is performed in a blinded fashion at the Outpatient Clinic or by telephone, by a stroke nurse certified in NIHSS and mRS assessment (http://nihss-english.trainingcampus.net/uas/modules/trees/windex.aspx; http://rankin-english.trainingcampus.net/uas/modules/trees/windex.aspx).

### Vascular outcome (recanalisation)

24-hour recanalisation is assessed by MRA (or CTA when MRA is not possible) by a neurologist and/or neuroradiologist blinded to the drug used and detailed clinical data [22]. Vascular patency is assessed using adapted Thrombolysis in Myocardial Infarction (TIMI) criteria [22]. Vessel occlusion status is defined as complete occlusion (TIMI 0), minimal flow (TIMI 1), partial flow (TIMI 2), or normal flow (TIMI 3). Major vessel recanalisation is defined as improvement in baseline to 24–36 h TIMI score of ≥2, or complete recanalisation.

### Brain imaging outcome (infarct)

24-hour infarct is assessed by 24-hour MR-DWI (or CT when MRI is not possible) by a neuroradiologist in a clinically blinded fashion. The diagnostic work-up is summarised in Table 1.

**Table 1:** **Assessment of patients with acute ischaemic stroke admitted** <**4**½ **hours**

**Timepoint → Procedure ↓**	**Baseline**	**+ 2 h (after Rp/)**	**+ 24 h (22–36 h)**	**+ 48 h (42–54 h)**	**Day 7 or earlier discharge**	**Day 90**
NIHSS score	X	X	X	X	X	
CT/CTA/MRI/MRA	X		X			
ECG	X					
Modified Rankin Scale (mRS)					X	X
Barthel Index (BI)					X	
Check Recurrent stroke/TIA						X
Check Acute coronary heart disease						X

### Safety outcome (bleedings and death)

Patients are monitored in the stroke unit (or ICU if standard) with NIHSS scoring at close intervals for 24–36 hours. Haemorrhagic transformation is assessed by 24-hour MRI (or CT when MRI is not possible) by a neuroradiologist blinded to all clinical data.

Haemorrhagic transformation (ECASS criteria) is categorised as:

•Haemorrhagic infarction 1 (HI1) = small petechiae along the margins of the infarct

•Haemorrhagic infarction 2 (HI2) = confluent petechiae within the infarcted area but no space-occupying effect

•Parenchymal haemorrhage (PH1) = blood clots in 30% or less of the infarcted area with some slight space-occupying effect

•Parenchymal haemorrhage (PH2) = blood clots in more than 30% of the infarcted area with substantial space-occupying effect

•Remote parenchymal haemorrhage (rPH) = bleeding outside the infarcted area

Symptomatic intracranial haemorrhage (SICH) is assessed as:

•local or remote parenchymal haemorrhage type 2 on the 24–36 h post-treatment imaging scan, combined with a neurological deterioration of 4 points or more on the NIHSS from baseline or from the lowest NIHSS value between baseline and 24 h, or leading to death (*SITS*-*MOST criteria*).

In order to facilitate comparison with published studies, SICH is also assessed:

•as any haemorrhage plus any neurological deterioration [NIHSS score ≥1] or that leads to death within 7 days (*NINDS criteria*; *Cochrane criteria*)

•blood at any site in the brain on the CT scan, clinical deterioration or adverse events indicating clinical worsening (drowsiness, increase of hemiparesis) or causing a increase in the NIHSS score of 4 or more points (*ECASS II criteria*)

•any apparently extravascular blood in the brain or within the cranium associated with clinical deterioration (defined by an increase in the NIHSS score of 4 or more points) or death, and that is identified as the predominant cause of the neurological deterioration (*ECASS III criteria*)

All procedural complications related to intraarterial embolectomy (groin hematoma, vessel dissection, vessel perforation, subarachnoid haemorrhage) are registered.

Death within 90 days is registered. Death is checked against the National Public Register.

### Study endpoints

The primary study endpoint is favourable functional outcome defined as mRS 0–1 at 90 days. Secondary study endpoints are: 1) Haemorrhagic transformation (haemorrhagic infarct/haematoma); 2) Symptomatic cerebral haemorrhage on CT 24–48 hours; 3) Major neurological improvement at 24 hours; 4) Recanalisation at 24–36 hours; 5) death.

### Safety monitoring

Safety monitoring is performed by an independent Data safety and monitoring committee (DSMC).

Intravenous thrombolysis: Periodic safety review is performed after each 40 enrolled patients (20 per group). Formal interim analysis is performed once a year. Formal safety “stopping rules” are applied for safety. Based on data from the literature on the frequency of SICH in trials or clinical series of thrombolysis, the overall stopping rule is set at a rate of SICH that exceeds 8% in any group, the stopping rule for tenecteplase 0.4 mg/kg is set at a 2% excess of SICH. In the event of SICH surpassing these limits, the trial will temporarily (pending review of the data) stop further patient accrual. To maintain blinding within the committee, the calculations will be performed by a statistical consultant, who is required to simply report whether the “stopping rule” is met (i.e., no disclosure of interim data if the stopping guideline is not met). If the DSMC finds unacceptable safety (bleedings/death) with tenecteplase, the tier 0.4 mg/kg will be stopped and the trial continued with a step-down to tenecteplase 0.25 mg/kg.

Intra-arterial embolectomy: Periodic safety review is performed after each 30 enrolled patients (15 per group). Based on data from the literature on the frequency of SICH in trials or clinical series of endovascular embolectomy, the overall stopping rule is set at a rate of SICH that exceeds 14% (2/15) in any group. In the event of SICH surpassing these limits, the trial will temporarily (pending review of the data) stop accrual of patients treated with endovascular embolectomy.

All suspected unexpected serious adverse reactions, which may be related to the study drug, will be reported to the Norwegian Medicines Agency and to the DSMC. All serious adverse events, i.e. bleedings and deaths, and other Adverse Events will be reported to the DSMC.

The overall rate of bleeding complications will be compared with historical data from NORSTROKE 2007–2011, the pooled analysis of IV alteplase trials [17], SITS-MOST [18,19], ECASS III [23] and series on thrombolysis in patients above 80 years of age [24]. The mortality rate at 7 days will be compared with historical data from NORSTROKE 2007–2011.

The over-all rate of bleeding complications will be compared with historical data from NORSTROKE 2007–2012 and from Stavanger University Hospital [25], Interventional Management of Stroke (IMS) I [26], IMS II [27] and IMS III [28].

### Safety outcome (futility)

If the trial shows no significant difference between the two treatment groups, the DSMC may stop the trial, if there also is a clear trend towards increased bleeding rate in one group.

### Statistical analysis

The primary analysis in NOR-TEST is to test whether tenecteplase is superior to alteplase. The primary analysis is an “intention to treat” analysis. The results will also be tested in a “per protocol” analysis. The data from the Wake-up-subset and Embolectomy-subset will be analysed separately and not as a part of the main NOR-TEST data set. Excluded patients admitted <4½ hours will also be analysed for comparison with thrombolysed patients and for inter-centre inclusion/exclusion differences (bias). Stratification according to age (≤80 vs. >80), baseline NIHSS (≤14 vs. >14) and time (0–3 h vs. 3-4½ h) will be performed.

### Ethics & data anonymity

The trial is performed in accordance with the European ‘Guidelines on Good Clinical Research Practice’ (Consolidated guideline, CPMP/ICH/135/95) and the Norwegian ‘Forskrift om klinisk utprøving av legemidler til menneske’ (FOR 2009-30-10).

NOR-TEST is reviewed and approved by the Regional Committee for Medical and Health Research Ethics (2011/2435), and The Norwegian Medicines Agency (12/01402). NOR-TEST is registered with EudraCT No 2011-005793-33 and in ClinicalTrials.gov (NCT01949948).

## Discussion

Despite indications of clinical superiority of tenecteplase compared to alteplase [1,9,12], tenecteplase in acute ischemic stroke has been studied only in small series with highly selected patients (visible clot, perfusion mismatch etc.) [9-11,29]. A large randomised controlled phase 3 trial has not been performed.

NOR-TEST is a pragmatic multi-centre phase 3 trial with logistic equivalent to the routine chain of treatment. NOR-TEST is therefore easily adopted by all hospitals, securing a high centre compliance and adherence to the protocol. All patients found eligible for standard alteplase treatment may be included. NOR-TEST will therefore apply tenecteplase to a general population of ischaemic stroke patients.

In patients receiving embolectomy, the hypothesised higher recanalisation rates of tenecteplase as compared to alteplase may benefit patients, as clots may partially or completely recanalise prior to the endovascular procedure.

There is no strong evidence that the highest dose of tenecteplase (0.4 mg/kg) increases the risk of haemorrhagic transformation [7,9,13]. In order to maximise the thrombolytic effect, NOR-TEST therefore is designed with a high tenecteplase dose together with careful safety monitoring of major bleedings.

A positive NOR-TEST result may give an impetus to implementing thrombolysis at a higher rate than today, and establish a more effective and less harmful treatment for all ischaemic stroke patients eligible for thrombolysis.

## Abbreviations

APTT: Activated Partial Thromboplastin Time; ASPECTS: Alberta Stroke Program Early CT score; BI: Barthel Index; CNS: Central Nervous System; CT: Computer Tomography; CTA: CT-angiography; ICU: Intensive Care Unit; MCA: Middle Cerebral Artery; MRI: Magnetic Resonance Imaging; mRS: modified Rankin Scale; NIHSS: National Institutes of Health Stroke Scale; NOR-TEST: Norwegian tenecteplase Stroke Trial; PAI: Plasminogen Activator Inhibitor; PROBE: Prospective Randomised, Open-label, Blinded endpoint; SICH: Symptomatic intracranial haemorrhage; SOP: Standard Operating Procedures; WUPS: Wake-up stroke.

## Competing interests

The study is not receiving funding/assistance from any commercial organizations.

## Authors’ contributions

LT obtained funding for the study. All authors contributed to the research design, the intervention, outcome measures and project management. NL and LT are principally responsible for the assessments, data-analysis and drafting of the manuscript. All authors critically reviewed the manuscript and approved the submitted version.

## Pre-publication history

The pre-publication history for this paper can be accessed here:

http://www.biomedcentral.com/1471-2377/14/106/prepub

## Supplementary Material

Additional file 1 Power calculations for excellent outcome (mRS 0-1) 90 days. Click here for file
