# Supplementary material for: The Norwegian tenecteplase stroke trial (NOR-TEST): randomised controlled trial of tenecteplase vs. alteplase in acute ischaemic stroke
Source: BMC Neurol. 2014 May 15;14:106. doi: 10.1186/1471-2377-14-106 (PMC4029902; doi:10.1186/1471-2377-14-106)

# Additional file 1. Power calculations for excellent outcome (mRS 0-1) 90 days

**Historical % 0-3 h % 3-4.5 h**

SITS (n=10231 / n=541) 40 41

Pooled alteplase (n=463) 42

Bergen NORSTROKE (n=134) 40

**pooled**

**Parsons 2012** (0-6^h^) (tenecteplase 0.25 + 0.1 mg/kg) **alteplase tenecteplase P value**

Excellent outcome (mRS 0-1) 90 days, n (%) 10 (40) 27 (54) 0.25

Good outcome (mRS 0-2) 90 days, n (%) 11 (44) 36 (72) 0.02

Any parenchymal hemorrhage 5 (20) 3 (6) 0.11

Symptomatic intracranial hemorrhage 3 (12) 2 (4) 0.33

**Selection:** Selection criteria enhanced the power of the study to detect a difference in efficacy.

One cannot extrapolate results to the majority of patients who are eligible for thrombolysis.

**Time window:** 0-6 hours weakens the results, but results are still similar to alteplase 0-4.5 hours.

**Dose response:** The higher dose of tenecteplase (0.25 mg/kg) was superior to the lower dose (0.1 mg/kg)

for all imaging and clinical efficacy outcomes, i.e. a dose-response.

**NOR-TEST compared with Parsons 2012**

Extending thrombolysis to all patients will dilute/reduce positive results compared with Parsons

Reducing time window 0-4.5 may increase the effect compared with Parsons

Increasing dose to 0.4 mg/kg will increase the effect compared with Parsons

**Basis for power calculation**

Excellent outcome at 90 days with alteplase (SITS) 41 %

Excellent outcome at 90 days with alteplase (Bergen NORSTROKE) 40 %

Excellent outcome at 90 days with tenecteplase (Parsons) 54 %

Excellent outcome at 90 days with alteplase (**NOR-TEST estimate**) 40 %

Excellent outcome at 90 days with tenecteplase (**NOR-TEST estimate**) 49 %

**NOR-TEST sample size**

NOR-TEST aims at detecting a 9 % higher percentage excellent outcome with tenecteplase vs. alteplase,

i.e. NOR-TEST total sample 954 patients (477 in each treatment group), see Figure S1

**Figure S1. Power calculation for superiority of tenecteplase vs. alteplase.**


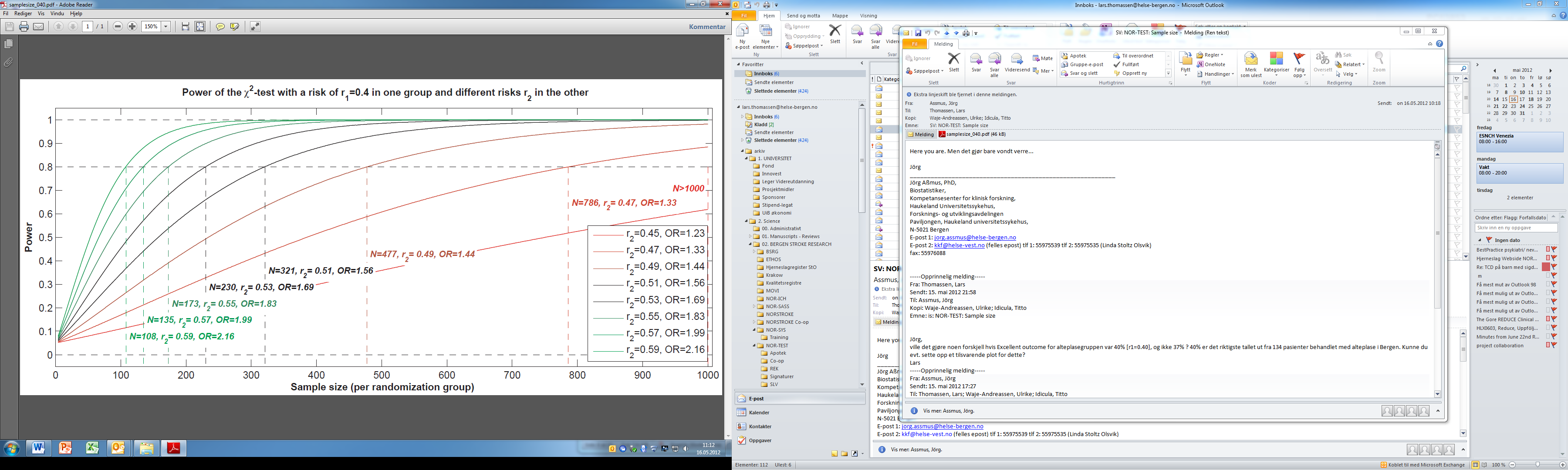

Supplement: Additional file 1 — Power calculations for excellent outcome (mRS 0-1) 90 days. [file 1471-2377-14-106-S1.doc]
